# Is Antimicrobial Photodynamic Therapy Effective as an Adjunct to Scaling and Root Planing in Patients with Chronic Periodontitis? A Systematic Review

**DOI:** 10.3390/biom7040079

**Published:** 2017-11-24

**Authors:** Betsy Joseph, Presanthila Janam, Subhash Narayanan, Sukumaran Anil

**Affiliations:** 1 Department of Periodontics and Community Dental Sciences, College of Dentistry, King Khalid University, Abha 62521, Saudi Arabia; bjoseph@kku.edu.sa; 2 Department of Periodontics, PMS College of Dental Sciences, Trivandrum, Kerala 695028, India; ksucod@gmail.com; 3 Sascan Meditech Private Limited, Centre for Innovation in Medical Electronics, BMS College of Engineering, Bangalore 560019, India; drsubashnarayanan@gmail.com; 4 Department of Periodontics, Saveetha Dental College and Hospitals, Saveetha University, Poonamallee High Road, Chennai 600077, India

**Keywords:** photodynamic therapy, bacterial biofilm, chronic periodontitis, photochemotherapy, systematic review

## Abstract

The aim of this systematic review was to investigate whether antimicrobial photodynamic therapy (aPDT) as either a primary mode of treatment or an adjunct to non-surgical treatment was more effective than scaling and root planing (SRP) alone in treating chronic periodontitis in terms of clinical attachment level (CAL) gain and probing depth (PD) reduction. The focused question was developed using the Patient, Intervention, Comparison, and Outcome (PICO) format, and two authors independently searched the Medline, EMBASE, Cochrane Library, Web of Science, Google Scholar, and Scopus databases for relevant studies from January 2008 to December 2016. Twenty studies included in this systematic review were randomized clinical trials (RCTs) or quasi-RCTs of aPDT compared to placebo, no intervention, or non-surgical treatment in an adult population. Basic study characteristics, photosensitizing agents and wavelengths used in aPDT, frequency of aPDT application, effect of aPDT on clinical parameters, antimicrobial effect of aPDT in chronic periodontitis, effect of immunological parameters following aPDT and patient-based outcome measures were collected from the studies. Although there was a wide range of heterogeneity in the included studied, they all indicated that aPDT has the potential to be an effective adjunct in the treatment of chronic periodontitis. Long-term, multicenter studies with larger sample sizes are needed before aPDT can be recommended as an effective treatment modality.

## 1. Introduction

The ultimate goal of periodontal therapy is to eliminate supragingival and subgingival plaque and arrest the progression of periodontal disease. Scaling and root planing (SRP) are considered as the gold standard for the treatment of chronic periodontitis. Although many studies have shown significant improvements following SRP, complete elimination of subgingival periodontal pathogens and irritants is not always possible [1,2]. Residual pockets during SRP present similar challenges and additional therapeutic approaches to achieving periodontal health are required. To improve the results of mechanical debridement, antibiotics are widely used [3,4]. Limitations of drug resistance associated with the use of local and systemic medications have led to the popularity of antimicrobial photodynamic therapy (aPDT) in the management of chronic periodontitis. aPDT was introduced in 1904 as the light-induced inactivation of cells, microorganisms or molecules. This treatment modality is based on the principle that a photoactivatable substance, called a photosensitizer, is activated by the light of a particular wavelength. The transfer of energy causes the formation of free radicals of singlet oxygen, which exert destructive action on bacteria and their products [5,6].

Even though the effects of photodynamic action have been known for a long time, interest in its practical use has increased only in the last few years. Because several studies had shown that killing both Gram-positive and Gram-negative bacteria is possible, Wilson’s group in London investigated different aspects of the application of aPDT in dentistry in vitro and in vivo [7,8,9]. In the presence of various types of photosensitizers, such as toluidine blue O and methylene blue, several periodontal pathogens are found to be susceptible to red lasers, which points to the fact that aPDT could be could be advantageous in periodontal therapy [10]. However, randomized controlled trials and systematic reviews have shown contrasting results regarding the efficacy of aPDT in chronic periodontitis [10,11,12,13,14,15,16]. Hence, the objective of the systematic review was to determine the effectiveness of aPDT as a primary mode or as an adjunct to non-surgical periodontal therapy.

## 2. Methodology

### 2.1. Search Strategy

The search strategy was based on the question “Is aPDT as either a primary mode of treatment or an adjunct to non-surgical treatment more effective than SRP alone in chronic periodontitis in terms of clinical attachment level (CAL) gain and probing depth (PD) reduction?”. This focused question was developed using the Patient, Intervention, Comparison, and Outcome (PICO) format [17]. Two authors independently searched the Medline, EMBASE, Cochrane Library, Web of Science, Google Scholar, and Scopus databases from January 2008 to December 2016 for relevant studies. The following terms in various combinations were used: bacteria, diode laser, lethal photosensitization, photodynamic inactivation, photodynamic antimicrobial chemotherapy, photodynamic therapy, and periodontitis.

### 2.2. Eligibility and Information Sources

**Figure 1:**
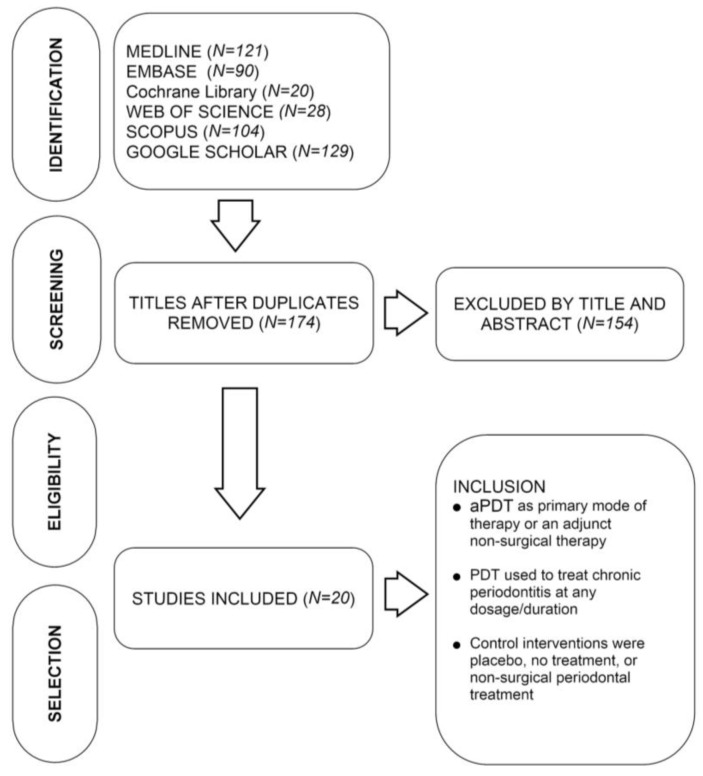
Decision tree showing the selection of articles included in the review. aPDT: antimicrobial photodynamic therapy; PDT: photodynamic therapy.

The 20 studies included in this systematic review were randomized clinical trials (RCTs) or quasi-RCTs of aPDT compared to placebo, no intervention, or non-surgical treatment in an adult population (Figure 1). In all studies, aPDT was either a primary mode of therapy or an adjunct to other non-surgical treatments, with CAL and/or PD as the primary outcome measures. Studies of aPDT used for the treatment of periodontitis at any dosage or duration were included. Eligible control interventions that were considered for this systematic review were placebo, no treatment, or non-surgical periodontal treatment (independent of or as adjunct therapy). Letters to the editor, short commentaries, and review articles were excluded.

### 2.3. Study Selection and Data Collection

To minimize the potential for reviewer bias, two blinded reviewers independently screened all titles and abstracts identified through electronic and manual searches. Disagreements regarding the inclusion or exclusion of studies were resolved by a discussion between the reviewers. Two reviewers used an extraction form to categorize the included articles in terms of patient demographic characteristics, presence of smokers, laser settings, and reported outcomes measures. The quality of the studies included in the systematic review was determined separately by two independent reviewers.

## 3. Results

### 3.1. Study Characteristics

The mean age of patients in the studies included in this review ranged from 39.6 years to 62.8 years. Studies that recruited patients with chronic periodontitis were included in this review. The characteristics of the included studies are shown in Table 1. Criteria of chronic periodontitis [12], severe periodontitis [18], pocket depth ≥5 mm [19,20,21,22], untreated periodontal pockets [12], pocket depth between 4 and 6 mm [23,24], pocket depth between 5 and 9 mm [25], and residual pockets during supportive periodontal therapy [13,26,27,28,29,30] were used. The presence of *Fusobacterium nucleatum* in localized chronic periodontitis was an inclusion criterion in one study [31]. Most of the studies included either single-rooted teeth or both single- and multi-rooted teeth, while two studies reported the effects of aPDT only in multi-rooted teeth [19,32]. Most of the remaining studies evaluated aPDT as both an adjunct to SRP in the management of chronic periodontitis and a monotherapy [24,26,27,30].

### 3.2. Study Design

The included studies were RCTs published between 2008 and 2015. Of the 20 studies included, six were parallel two-arm [12,13,19,23,30,31], one was parallel three-arm [27], one was a split-mouth four-arm [24], five were split-mouth three-arm [20,25,26,28,29], and six were split-mouth two-arm studies [11,18,21,22,32,34]. In vitro studies, in vivo studies with animals, and clinical reports were excluded. In split-mouth RCTs, each subject is its own control, and most of the variability of outcome among patients is removed from the intervention effect estimate. Bias may be induced [35] due to the “spilling” of the effects of one therapy from one site to another. When evaluating the results of aPDT in split-mouth studies, the paired nature of the data must be taken into account [36]. Because every subject receives each intervention, the split-mouth design may be better suited to studies that determine patient preferences.

### 3.3. Sample Size and Calculation

The sample sizes ranged from 15 to 90, with all studies but four having more female participants than males [12,21,27,28]. Of the 29 studies, 13 reported on the calculation of sample size with the power of the study set at or above 80% (81–86). Among these, nine studies reported that sample size was calculated based on probing depth, while five used CAL as the primary outcome. Significant changes reported in the studies must be interpreted with caution because to detect even a moderate change, at least 40 patients may be needed in one arm of the treatment. Generally, 20% is added to compensate for any drop-outs. The sample size is calculated as the number of patients needed for one arm, but few studies adhered to that. The lack of sample size calculation and reported methods of randomization were the main methodological issues noted. The reasons for drop-outs were also not specified in most of the studies.

### 3.4. Blinding

Blinding is done in order to decrease or hide the information regarding the type of intervention given to a particular participant so that outcomes and assessments of outcomes are unaffected. In this review, eight studies were double-blinded, eight were single-blinded, and four did not mention the type of blinding. The examiner and biostatistician were blinded in these trials. Details are provided in Table 2.

### 3.5. Smokers

Smoking has been associated with an increased occurrence of periodontitis. Table 1 shows that four of the 19 studies included both smokers and non-smokers [13,27,28,29]. Of these, two showed an improved reduction in PD compared with a control group [27,29]. When interpreting the results, it should be noted that an intention-to-treat analysis was not mentioned in a few studies. Studies by Cappuyns et al. [29] and Chondros et al. [13] included smokers and showed microbiological improvement, while immunological profiles were found to be improved in the study by Campanile et al. [27]. In this study, although detection frequencies of periodontal pathogens did not change significantly from baseline to month 3 or 6 in any group, significant overall decreases were observed from baseline to month 6 for C-reactive protein, serum amyloid A, fibrinogen, procalcitonin, and α-2 macroglobulin. Single or double episodes of aPDT showed some additional benefit over ultrasonic instrumentation alone.

### 3.6. Photosensitizing Agents and Wavelengths Used in aPDT

Antimicrobial photodynamic therapy has been applied using various combinations of lasers and photosensitizing (PS) agents. Methylene blue (3,7-bis(dimethyl-amino) phenazathionium chloride tetramethylthionine chloride)) was the most commonly used photosensitizing dye in the clinical trials. Toluidine blue O was also reported [24,25,30]. Table 3 shows the laser settings of the included studies. Toluidine blue O and methylene blue have similar chemical and physicochemical characteristics and have been used previously to detect mucosal tumors or atypical epithelia because they do not stain normal mucosa. They are the PS agents of choice for aPDT because they have a pronounced cationic charge that helps them bind to the outer membrane of Gram-negative bacteria and penetrate bacterial cells, thereby demonstrating a high degree of selectivity for killing microorganisms compared with host mammalian cells [37,38]. These dyes have been used in various concentrations (1 mg/mL to 10 mg/mL) with a residence time of 1 to 5 min in the periodontal pocket. Only two studies reported the quantity of PS used, which was either 0.2 mL methylene blue or 1 mL [25,27]. After a resident period of 1 to 3 min, excess PS was flushed off so that it would not act as an optical shield during laser irradiation [23].

Diode lasers between the wavelength of 635 nm and 670 nm were commonly used, although wavelengths of 808 nm [20] and 940 nm [22] were used in some studies. Optical fiber applicators with various diameters, ranging from 200 μm to 750 μm, were used. The laser application time was generally 60 s, although application times of 30 s [22] and 150 s [25] were also reported. Laser energy between 3 J/cm^2^ and 320 J/cm^2^ was used. The large differences in these laser parameters make it impossible to compare the results reported by various studies.

Wavelength and energy density are both important factors in the efficacy of lasers, and wavelength and optimal dose with an appropriate photosensitizer are practical variables in the bactericidal process [40]. It appears that differences in these factors led to different results.

### 3.7. Frequency of aPDT Application

Most of the studies included used a single session of aPDT, but some [22,24,27] used multiple applications (Table 3). The results of these studies on the effects of aPDT in terms of pocket depth reduction and clinical attachment gain differ. Two of the three studies that applied aPDT more than once showed improvements in clinical parameters compared to the control group [22,27]. These two studies also showed improvements in both immunological and microbiological parameters, which could be related to the repeated use of aPDT. De Paula Eduardo et al. [41] found that the application of multiple laser treatments is more effective than a single treatment. Multiple uses of aPDT during the first weeks of treatment may have increased the antimicrobial effect. One study [42] mentioned that the short time of exposure to light can be one reason for aPDT’s lack of effect.

### 3.8. Effect of aPDT on Clinical Parameters

Primary outcome measures were probing pocket depth reduction and CAL gain, which were defined as the difference between PD and CAL levels, respectively, at baseline and at the end of the follow-up period [33,43]. A change in bleeding on probing was the most common secondary outcome among the clinical parameters [44]. Microbiologic and immunologic changes, any adverse effect reported by the authors, and patient-based outcome measures were also studied [45]. All studies included in this review evaluated the effect of aPDT on clinical parameters (Table 2). Changes in PD and CAL were reported in all studies. While nine studies [11,21,22,23,27,31,32,33,34] reported improvements in PD following aPDT, the remaining studies did not report any additional benefit of aPDT compared to SRP [12,13,18,19,20,24,25,26,28,29,30]. These outcome parameters were re-evaluated at various time intervals, ranging from two weeks to one year. The results must be interpreted carefully as various factors could affect the results of aPDT. Pressure-calibrated probes [11] and examiner calibrations [12,13,18,19,20,23,24,25,26,32] were used in several studies for standardization.

### 3.9. Antimicrobial Effect of aPDT in Chronic Periodontitis

Pathogenic periodontal microorganisms such as *Porphyromonas gingivalis*, *Prevotella intermedia*, *Fusobacterium nucleatum*, and *Parvimonas micra* have been destroyed by photodynamic action [46]. The reduction of the biological activities of the key virulence factors, such as lipopolysaccharide and proteases, may act as an additional benefit. In comparison, microbiological changes were not well evaluated, as shown in Table 4. Only eight of the 20 studies reported microbiological changes. Six of these [13,19,25,29,31,33] reported a reduction in periodontal pathogens in the test group at various time intervals. Clinical improvements along with microbiological changes were observed in two studies [29,31]. PCR was used in all studies but one to detect the microbiological changes; in that study, RNA probes were used [29]. Sigusch et al. [31] studied *Fusobacterium nucleatum*-infected chronic periodontitis patients. Other organisms that were evaluated included *Eubacteriumnodatum* [13], *Porphyromonas gingivalis*, *Aggregatibacter actinomycetemcomitans*, *Tannerella forsythia*, *Treponema denticola*, *Prevotella intermedia*, *Parvimonasmicra*, *Fusobacterium nucleatum*, *Eikenellacorrodens*, and *Capnocytophaga* sp. [19,25,29]. Significant reductions were observed in the levels of *F. nucleatum* [13,31], *E. nodatum* [13], *P. gingivalis*, *T. forsythia*, *T. denticola* [29], and *E. corrodens* [25]. The mechanism through which aPDT kills microorganisms such as *P. gingivalis* and *F. nucleatum* has been established [47]. The lethal photosensitization of these microorganisms must involve changes in membranes and/or plasma membrane proteins and DNA damage mediated by singlet oxygen [47].

### 3.10. Effect of Immunological Parameters Following aPDT

Five studies reported improvements in immunological parameters [19,22,26,27,28], as shown in Table 4. Improvements in levels of Interleukin (IL)-1b and IL-6, IL-4 [22], C-reactive protein, serum amyloid A, fibrinogen, procalcitonin, α-2 macroglobulin [27], granulocyte macrophage colony-stimulating factor (GM-CSF), interferon, IL-8 [19] were reported. Among these, only one study also showed clinical improvement [27]. It should be noted that in this study, aPDT was administered twice a week. A significant overall decrease was observed from baseline to month 6 for C-reactive protein, serum amyloid A, fibrinogen, procalcitonin, and α-2 macroglobulin. When looking at the groups separately, C-reactive protein was significantly lower only when the laser had been activated twice. Other differences between groups were not significant.

### 3.11. Patient-Based Outcome Measures Reported in the Studies

Traditional measures of health outcomes do not capture patients’ perspectives of the disease, and therefore, patient-based outcomes were identified as a research priority [48]. Table 3 shows that only four studies reported patients’ perspectives of aPDT. Three of these studies [26,27,29] report pain perceptions of patients during the procedure using the visual analogue scale (VAS). In the study by Kolbe et al. [26] there were no differences in VAS scores between protocols for any of the parameters described. SRP-treated sites required significantly more anesthesia than did those treated with other therapies. Cappuyns et al. [29] reported that scores > 40 mm were similar in all treatment groups, and the tendency for more frequent VAS scores > 30 mm after SRP did not reach statistical significance. The total treatment time per quadrant, the use of local anesthetics, and the sequence of the treatments had no significant impact. Campanile et al. [27] reported patient discomfort following aPDT in periodontitis. It was found that only two of 27 patients being treated for residual periodontal pockets reported pain > 40 mm on a 0–100 mm VAS scale. Neither case was directly related to the aPDT procedure. One was due to tabmechanical debridement and another to a feeling of illumination in the eye when a pocket mesial of a first maxillary molar was irradiated. Halitosis as perceived by patients following the treatment was reported in one study [23]. Halitosis as detected by the hand-over-mouth technique was found to be improved after one month of treatment and did not persist beyond that time. There is increasing evidence that this treatment modality enhances wound healing following mechanical debridement by decontamination and tissue stimulation [49]. The latest studies have also shown that a combination of SRP and PDT results in substantially higher short-term clinical improvements, evidenced by probing depth or bleeding on probing reductions compared with SRP alone [49], including short-term reduction in *A. actinomycetemcomitans* levels in treating residual pockets after 3 months [46].

## 4. Conclusions

aPDT is emerging as a beneficial therapeutic option in the treatment of periodontitis. The results of many studies, if not all, indicate that aPDT along with SRP has a clear-cut advantage in the treatment of periodontitis. The additional benefits of aPDT in terms of clinical, microbiological, immunological, and patient-based outcomes are definitely encouraging and, hence, should be included in the routine treatment protocol of patients with periodontitis. Although there was a wide range of heterogeneity in the included studies, they all indicated that aPDT has the potential to be an effective adjunct in the treatment of chronic periodontitis. Long-term, multicenter studies with larger sample sizes are needed before aPDT can be recommended as an effect treatment modality.

## Figures and Tables

**Table 1:** Studies on aPDT in chronic periodontitis with clinical attachment level (CAL) or probing depth (PD) as the primary outcome measures.

Author Country	Sample Size (Male/Female) and Mean Age	Study design; Power of Study; Case Allotment	Outcome Measured	Treatment Arms	Conclusion
Kolbe et al. [26] Brazil	22 (10/12) 48.52 y	Split-mouth 83% (CAL) Computer-generated	Clinical, Microbiology (polymerase chain reaction(PCR)); Pain perception (Visual Analogue Scale (VAS))	Scaling and Root Planing (SRP) aPDT Photosensitizer	All therapies promoted similar improvements in clinical parameters. The aPDT protocol presented inferior frequency of *Porphyromonas gingivalis* at three months when compared with the other therapies. aPDT as an exclusive therapy may be considered a non-invasive alternative for treating residual pockets and offers advantages in the modulation of cytokines.
Carvalho et al. [33] Brazil	34 (21/13) 48 y	Parallel	Pocket probing depth (PPD), CAL, bleeding on probing (BoP) and plaque index (PI)	SRP aPDT	Both treatments resulted in significant clinical improvement in patients with residual periodontal pockets. We did not find any additional significant benefit of PDT in terms of PPD, CAL, BoP, or pathogen level reduction.
Betsy et al. [23] India	90 (39/51) 39.6 y	Parallel 80% (PD) Tippet’s 2-digit number table	Clinical and halitosis as perceived by patient	SRP SRP + aPDT	PD improved after three months and halitosis after one month. Statistically significant improvements in the gingival index and gingival bleeding index were observed for the test group after two weeks and one month of aPDT, respectively. aPDT is a beneficial adjunct to SRP in the non-surgical treatment and management of chronic periodontitis in the short term.
Luchesi et al. [19] Brazil	37 50.5 y	Parallel 86% (CAL) Computer-generated	Clinical, Microbiology (PCR); Immunology (granulocyte-macrophage colony-stimulating factor (GM-CSF), interferon (IFN), Interleukin (IL)-6 and Interleukin-8 levels)	SRP + aPDT SRP + non-activated light (only PS)	Clinical parameters improved after both therapies. Did not promote clinical benefits for class II furcations; however, there were advantages in terms of the local levels of cytokines and periodontopathogens reduction.
Dilsiz et al. [20] Turkey	24 (10/14) 40.7 y	Split-mouth Computer-generated		SRP SRP + aPDT SRP + KTP	Improvement in PD and CAL gain following treatment. Additional use of potassium titanyl phosphate (KTP) laser was found to be better in improving clinical parameters than conventional periodontal therapy of deeper pockets.
Alwaeli et al. [32] Malaysia	21 (7/14) 40.9 y	Split-mouth Computer-generated		SRP SRP + aPDT	Significant improvement in all evaluated clinical parameters for at least one year. There were significantly greater reductions and gains for SRP + aPDT than for SRP at all three-time points. aPDT as an adjunctive therapy to SRP represents a promising therapeutic concept for persistent periodontitis.
Campanile et al. [27] Switzerland	27 (14/13) 62.8 y	Parallel Smokers included 80% (PD) Computer-generated	Clinical, Microbiology (PCR); Pain perception (VAS); Immunology (C-reactive protein, Serum amyloid A, fibrinogen, procalcitonin, and α-2 macroglobulin)	aPDT twice in one week aPDT once Sham without active light	Significant PD and BoP reduction after three months when aPDT was administered twice a week. C-reactive protein was significantly lower only when the laser had been activated twice.
Bassir et al. [24] USA	16 (8/8) 50.3 y	Split-mouth 80% (CAL) Computer-generated	Clinical	LED PS aPDT SRP	No additional benefit was noticed with administration of photoactivated disinfection (PAD) using LED in patients with moderate to severe chronic periodontitis.
Campos et al. [34] Brazil	15 (8/7) 48.1 y	Split-mouth 80% (PD) Computer-generated	Clinical	SRP SRP + aPDT	aPDT as an adjunctive to mechanical debridement demonstrated additional clinical benefits for residual pockets in single-rooted teeth and may be an alternative therapeutic strategy in supportive periodontal maintenance.
Balata et al. [18] Brazil	22 (8/14) 43.18 y	Split-mouth 80% (CAL) Coin toss	Clinical	SRP SRP + aPDT	Both approaches resulted in significant clinical improvement in the treatment of severe chronic periodontitis. aPDT did not provide any additional benefit.
Barekdar et al. [21] Germany	22 (12/10) 59.3 y	Split-mouth	Clinical	SRP SRP + aPDT	A greater reduction of the PD was achieved by a combination of SRP/aPDT; therefore, aPDT is suitable as an adjuvant therapy.
Giannopoulou et al. [28] Switzerland	32 (23/9) 52 y	Split-mouth Smokers included 80% (PD) Computer-generated	Clinical, Immunology (IL-17, basic fibroblast growth factor, granulocyte-macrophage colony-stimulating factor (GCSF), macrophage inflammatory protein (MIP))	SRP Diode laser aPDT	No significant differences were observed among the three treatment modalities at any time point for any biochemical parameter or enhanced expression of inflammatory mediators.
Cappuyns et al. [29] Switzerland	32 (23/9) 52 y	Split-mouth Smokers included 80% (PD) Computer-generated	Clinical, Microbiology (RNA probes); Pain perception (VAS)	SRP Diode laser aPDT	At the end of six months, statistically significant PD and BoP reductions were recorded. Frequencies of three periodontal pathogens were significantly lower in groups with aPDT- and SRP-treated than in diode soft laser-treated quadrants after 14 days. However, the same was not noticed at the end of two and six months. aPDT resulted in a reduction in the number of pockets after six months.
Lui et al. [22] Hong Kong	24 (10/14) 50 y	Split-mouth	Clinical, Immunology (IL-1b levels in gingival crevicular fluid)	SRP SRP + one course of low level laser therapy (LLT) and aPDT within 5 days	The test teeth achieved greater reductions in the percentage of sites with bleeding on probing and in mean probing depth at one month compared with the control teeth and also greater reduction of interleukin (IL)-1b levels in gingival crevicular fluid at 1 week than did the control sites. No significant differences in periodontal parameters were found between the test and control teeth at three months.
Theodoro et al. [25] Brazil	33 (12/21) 43.12 y	Split-mouth 81% (CAL) Computer-generated	Clinical, Microbiology (PCR)	SRP SRP + Toluidine Blue O (TBO) SRP + aPDT	All treatment groups showed an improvement in all clinical parameters and a significant reduction in the proportion of sites positive for periodontopathogens at 60, 90, and 180 days compared to baseline. None of the periodontal parameters showed a significant difference among the groups. At 180 days, aPDT treatment led to a significant reduction in the percentage of sites positive for all bacteria compared to SRP alone.
Sigush et al. [31] Germany	24 (7/17) 42.7 y	Parallel Drawing lots	Clinical, Microbiology (PCR)	SRP + PS SRP + aPDT	Significant reductions in reddening, BoP, and mean PD and CAL were observed during the observation period and with respect to controls. Appropriate to reduce periodontal inflammation and to successfully treat infection with *Fusobacterium nucleatum*.
Ruhling et al. [30] Germany	60 48 y	Parallel 80% (PD) Computer-generated		SRP aPDT	aPDT was not found to be better than routine mechanical debridement in the management of persistent pockets, but still maybe considered a valuable therapeutic option.
Christodoulides et al. [12] Germany	24 (13/11) 45 y	Parallel 80% (PD) Coin toss		SRP SRP + aPDT	Additional application of a single episode of aPDT to SRP failed to result in an additional improvement in terms of PD reduction and CAL gain, but resulted in a significantly higher reduction in bleeding scores compared to SRP alone.
Braun et al. [11] Germany	20 (9/11) 46.6 y	Split-mouth		SRP SRP + aPDT	Improvement in clinical parameters with the use of adjunctive aPDT as compared to subgingival debridement.
Chondros et al. [13] Germany	24 (10/14) 49.3 y	Parallel 80% (PD) Coin toss	Clinical, Microbiology (PCR)	SRP SRP + aPDT	Additional application of a single episode of aPDT to SRP failed to result in additional improvement. Significantly higher reduction of bleeding scores in test group. At three months and six months, a statistically significantly higher improvement of BoP was found in the test group. At three months after therapy, the microbiological analysis showed a statistically significant reduction of *F.nucleatum* and *Eubacterium nodatum* in the test group.

**Table 2:** The sample size and method used to derive samples in the selected studies.

Author Country	Sample Size (Male/Female)	Power of the Study	Type of Randomization	Type of Blinding	Case Allotment	Whether Intention-to-Treat (ITT) Analysis Done
Kolbe et al. [26] Brazil	22 (10/12)	83% (CAL)	Not mentioned	Double-blinded	Computer-generated	Yes
Carvalho et al. [33] Brazil	34 (21/13)	90% (CAL)	Block randomization (size = 4)	Double -blinded	Computer-generated	Yes
Betsy et al. [23] India	90 (39/51)	80% (PD)	Block randomization (size = 4)	Double-blinded	Tippet’s 2-digit number table	Yes
Luchesi et al. [19] Brazil	37	86% (CAL)	Not mentioned	Double-blinded	Computer-generated	Yes
Dilsiz et al. [20] Turkey	24 (10/14)	Not given	Not mentioned	Double-blinded	Computer-generated	Yes
Alwaeli et al. [32] Malaysia	21 (7/14)	Not given	Not mentioned	Double-blinded	Computer-generated	No
Campanile et al. [27] Switzerland	27 (14/13)	80% (PD)	Not mentioned	Single-blinded	Computer-generated	No
Bassir et al. [24] USA	16 (8/8)	80% (PD)	Block randomization (size = 1)	Double-blinded	Computer-generated	Not mentioned
Campos et al. [34] Brazil	15	80% (PD)	Not mentioned	Double-blinded	Computer-generated	Not mentioned
Balata et al. [18] Brazil	22 (8/14)	80% (CAL)	Not mentioned	Not given	Coin toss	Yes
Barekdar et al. [21] Germany	22 (12/10)	Not mentioned	Not mentioned	Single-blinded	Not mentioned	Yes
Giannopoulou et al. [28] Switzerland	32 (23/9)	80% (PD)	Not mentioned	Not mentioned	Computer-generated	No
Cappuyns et al. [29] Switzerland	32 (23/9)	80% (PD)	Not mentioned	Single-blinded	Computer-generated	No
Lui et al. [22] Hong Kong	24 (10/14)	Not mentioned	Not mentioned	Single-blinded	Not mentioned	Yes
Theodoro et al. [25] Brazil	33 (12/21)	81% (CAL)	Not mentioned	Single-blinded	Computer-generated	Yes
Sigush et al. [31] Germany	24 (7/17)	Not mentioned	Not mentioned	Not given	Drawing lots	Not mentioned
Ruhing et al. [30] Germany	60	80% (PD)	Not mentioned	Single-blinded	Computer-generated	No
Christodoulides et al. [12] Germany	24 (13/11)	80% (PD)	Not mentioned	Not given	Coin toss	Yes
Braun et al. [11] Germany	20 (9/11)	Not mentioned	Not mentioned	Single-blinded	Not mentioned	Yes
Chondros et al. [13] Germany	24 (10/14)	80% (PD)	Not mentioned	Single-blinded	Coin toss	Yes

**Table 3:** Laser parameters of the included studies.

Author Country	Photosensitizer Concentration	Resident Time of Photosensitizer	Laser Application Time	Laser Wavelength	Laser Output	Fiber Optic Tip Diameter	Laser Energy
Kolbe et al. [26] Brazil	Methylene blue 10 mg/mL	1 min	1 min	660 nm	60 mw/cm^2^	Not mentioned	129 J
Carvalho et al. [33] Brazil	Methylene blue 0.01%	5 min	1 min	660 nm	40 mw/cm^2^	Not mentioned	90 J
Betsy et al. [23] India	Methylene blue 10 mg/mL	3 min	1 min	655 nm	1 W/cm^2^	200 μm	Not mentioned
Luchesi et al. [19] Brazil	Methylene blue 10 mg/mL	1 min	1 min	660 nm	60 mw/cm^2^	600 μm	129 J
Dilsiz et al. [20] Turkey	Methylene blue (25 g) 1%	3 min	1 min	808 nm	100 mw/cm^2^	300 μm	6 J
Alwaeli et al. [32] Malaysia	Phenothiazine chloride	1 min	1 min	660 nm	100 mw/cm^2^	Not mentioned	Not mentioned
Campanile et al. [27] Switzerland	Methylene blue	1 min	1 min	670 nm	280 mw/cm^2^	Not mentioned	Not mentioned
Balata et al. [18] Brazil	Methylene blue 0.01%	2 min	1 min	660 nm	100 mw/cm^2^	Not mentioned	320 J
Bassir et al. [24] USA	Toluidine blue O 0.1 mg/mL	3 min	1 min	635 nm	2 W/cm^2^	Not mentioned	Not mentioned
Barekdar et al. [21] Germany	Methylene blue 0.01%	2 min	1 min	670 nm	150 mw/cm^2^	600 μm	Not mentioned
Giannelli et al. [39] Italy	Methylene blue 0.03%	5 min	1 min	635 nm	100 mw/cm^2^	600 μm	3.8 J
Giannopoulou et al. [28] Switzerland	Phenothiazine chloride; 100 μg/mL	3 min	1 min	660 nm	100 mw/cm^2^	750 μm	3 J
Cappuyns et al. [29] Switzerland	Phenothiazine chloride; 100 μg/mL	1 min	1 min	660 nm	40 mw/cm^2^	Not mentioned	Not mentioned
Lui et al. [22] Hong Kong	Methylene blue 1%	3 min	30 s	940 nm	1.5 W/cm^2^	Not mentioned	4 J
Theodoro et al. [25] Brazil	Toluidine blue O 100 μg/mL	1 min	150 s	660 nm	400 mw/cm^2^	Not mentioned	Not mentioned
Sigush et al. [31] Germany	Phenothiazine	1 min	1 min	660 nm	60 mw/cm^2^	0.6 mm	Not mentioned
Ruhing et al. [30] Germany	Tolonium chloride 5%	Not mentioned	1 min	635 nm	100 mw/cm^2^	Not mentioned	Not mentioned
Christodoulides et al. [12] Germany	Phenothiazine	3 min	1 min	670 nm	75 mw/cm^2^	Not mentioned	Not mentioned
Braun et al. [11] Germany	Phenothiazine	3 min	1 min	660 nm	100 mw/cm^2^	Not mentioned	Not mentioned
Chondros et al. [13] Germany	Phenothiazine	Not mentioned	1 min	670 nm	75 mw/cm^2^	Not mentioned	Not mentioned

**Table 4:** Studies reporting microbiologic, immunologic, and patient-based outcomes along with clinical parameters.

Author Country	Sample Size	Outcome Measured	Conclusions
Kolbe et al. [26] Brazil	22	Microbiology(PCR); Pain perception (VAS)	Similar improvements noticed in clinical parameters with all treatments. PDT protocol presented inferior frequency of *P. gingivalis* at three months when compared with the other therapies. aPDT as an exclusive therapy may be considered a non-invasive alternative for treating residual pockets, offering advantages in the modulation of cytokines.
Carvalho et al. [33] Brazil	34	Microbiology(PCR); Pocket probing depth (PPD), CAL, BoP and PI	All treatments resulted in significant clinical improvement in patients with residual periodontal pockets. PDT failed to show superior clinical results and pathogen load reduction in persistent pockets, compared to supragingival plaque control.
Betsy et al. [23] India	90	Halitosis as perceived by patient	Changes in PD after three months and halitosis after one month. Gingival index and gingival bleeding index improved significantly in the test group after two weeks and one month of aPDT. As an adjunct to SRP, aPDT shows effectiveness in the short term for managing chronic periodontitis.
Luchesi et al. [19] Brazil	37	Microbiology (PCR); Immunology (GM-CSF, IFN-c, IL-6 and IL-8 levels)	Clinical parameters improved after both therapies. At six months, real-time PCR evaluation showed a decrease in *P. gingivalis* and *Tannerella forsythia* only in the PDT group, with no inter-group differences. IL-4 and IL-10 levels increased in both groups at six months. GM-CSF, IL-8, IL-1b and IL-6 levels decreased only in the PDT group after three months. At three months, inter-group analyses showed that GM-CSF, IFN-c, IL-6 and IL-8 levels were lower in the PDT group. At six months, lower IL-1b levels were also observed in the PDT group. Did not promote clinical benefits for class II furcations.
Campanile et al. [27] Switzerland	27	Microbiology (PCR); Pain perception (VAS); Immunology (C-reactive protein, Serum amyloid A, fibrinogen, procalcitonin, and α-2 macroglobulin)	Detection frequencies of the studied microorganisms at >1000 and >100,000 cells/mL did not change significantly from baseline to months 3 or 6 in any group. Significant PD and BoP reduction after three months when aPDT given twice a week. C-reactive protein was significantly lower only if the laser had been activated twice.
Cappuyns et al. [29] Switzerland	32	Microbiology (RNA probes); Pain perception (VAS)	Statistically significant PD and BoP reduction was seen at six months. Frequencies of three microorganisms were significantly lower in aPDT- and SRP-treated than in diode soft laser-treated quadrants after 14 days, but not at months 2 and 6. aPDT resulted in fewer residual pockets after six months.
Giannopoulou et al. [28] Switzerland	32	Immunology (IL-17, basic fibroblast growth factor, granulocyte colony-stimulating factor, and macrophage inflammatory protein 1-a)	No significant differences were observed among the three treatment modalities at any time point for any biochemical parameter or enhanced expression of inflammatory mediators.
Theodoro et al. [25] Brazil	33	Microbiology (PCR)	All treatment groups showed an improvement in all clinical parameters, and a significant reduction in the proportion of sites positive for periodontopathogens at 60, 90, and 180 d compared to the baseline. None of the periodontal parameters showed a significant difference among the groups. At 180 days, PDT treatment led to a significant reduction in the percentage of sites positive for all bacteria compared to SRP alone.
Lui et al. [22] Hong Kong	24	Immunology (IL-1b levels in gingival crevicular fluid)	A significant decrease in gingival crevicular fluid volume was observed in both groups at one week, with a further decrease at one month in the test sites. The test sites showed a greater reduction of IL-1b levels in gingival crevicular fluid at one week than the control sites. No significant differences in periodontal parameters were found between the test and control teeth at three months.
Sigush et al. [31] Germany	24	Microbiology (PCR)	BoP, mean PD, and mean CAL showed improvement in the test group as compared to controls. aPDT may be used to manage periodontal inflammation and infection with *F. nucleatum*.
Chondros et al. [13] Germany	24	Microbiology (PCR)	Application of a single episode of aPDT to SRP failed to result in an additional improvement. Significantly higher reduction of bleeding scores in test group. At three and six months, a statistically significantly higher improvement of BoP was found in the test group. At three months after therapy, the microbiological analysis showed a statistically significant reduction of *F. nucleatum* and *E. nodatum* in the test group.
